# Neurometabolic Correlates of Reactive and Proactive Motor Inhibition in Young and Older Adults: Evidence from Multiple Regional ^1^H-MR Spectroscopy

**DOI:** 10.1093/texcom/tgaa028

**Published:** 2020-06-27

**Authors:** Akila Weerasekera, Oron Levin, Amanda Clauwaert, Kirstin-Friederike Heise, Lize Hermans, Ronald Peeters, Dante Mantini, Koen Cuypers, Inge Leunissen, Uwe Himmelreich, Stephan P Swinnen

**Affiliations:** Movement Control & Neuroplasticity Research Group, Department of Movement Sciences, Group Biomedical Sciences, KU Leuven, 3001, Heverlee, Belgium; Biomedical MRI Unit, Department of Imaging and Pathology, Group Biomedical Sciences, KU Leuven, 3000, Leuven, Belgium; A.A. Martinos Center for Biomedical Imaging, Massachusetts General Hospital, Harvard Medical School (MGH/HMS), Boston, 02129, MA, USA; Movement Control & Neuroplasticity Research Group, Department of Movement Sciences, Group Biomedical Sciences, KU Leuven, 3001, Heverlee, Belgium; Movement Control & Neuroplasticity Research Group, Department of Movement Sciences, Group Biomedical Sciences, KU Leuven, 3001, Heverlee, Belgium; Movement Control & Neuroplasticity Research Group, Department of Movement Sciences, Group Biomedical Sciences, KU Leuven, 3001, Heverlee, Belgium; Movement Control & Neuroplasticity Research Group, Department of Movement Sciences, Group Biomedical Sciences, KU Leuven, 3001, Heverlee, Belgium; Department of Radiology, University Hospitals KU Leuven, 3000, Leuven, Belgium; Movement Control & Neuroplasticity Research Group, Department of Movement Sciences, Group Biomedical Sciences, KU Leuven, 3001, Heverlee, Belgium; Brain Imaging and Neural Dynamics Research Group, IRCCS San Camillo Hospital, 30126, Venice, Italy; Movement Control & Neuroplasticity Research Group, Department of Movement Sciences, Group Biomedical Sciences, KU Leuven, 3001, Heverlee, Belgium; REVAL Research Institute, Faculty of Rehabilitation Sciences, Hasselt University, 3590, Diepenbeek, Belgium; Movement Control & Neuroplasticity Research Group, Department of Movement Sciences, Group Biomedical Sciences, KU Leuven, 3001, Heverlee, Belgium; Department of Cognitive Neuroscience, Faculty of Psychology and Neuroscience, Maastricht University, 6229 ER, Maastricht, The Netherlands; Biomedical MRI Unit, Department of Imaging and Pathology, Group Biomedical Sciences, KU Leuven, 3000, Leuven, Belgium; Movement Control & Neuroplasticity Research Group, Department of Movement Sciences, Group Biomedical Sciences, KU Leuven, 3001, Heverlee, Belgium; Leuven Brain Institute (KU Leuven-LBI), 3000, Leuven, Belgium

**Keywords:** aging, glutamate, myo-inositol, *N*-acetylaspartate, prefrontal-striatal pathways

## Abstract

Suboptimal inhibitory control is a major factor contributing to motor/cognitive deficits in older age and pathology. Here, we provide novel insights into the neurochemical biomarkers of inhibitory control in healthy young and older adults and highlight putative neurometabolic correlates of deficient inhibitory functions in normal aging. Age-related alterations in levels of glutamate–glutamine complex (Glx), *N*-acetylaspartate (NAA), choline (Cho), and myo-inositol (mIns) were assessed in the right inferior frontal gyrus (RIFG), pre-supplementary motor area (preSMA), bilateral sensorimotor cortex (SM1), bilateral striatum (STR), and occipital cortex (OCC) with proton magnetic resonance spectroscopy (^1^H-MRS). Data were collected from 30 young (age range 18–34 years) and 29 older (age range 60–74 years) adults. Associations between age-related changes in the levels of these metabolites and performance measures or reactive/proactive inhibition were examined for each age group. Glx levels in the right striatum and preSMA were associated with more efficient proactive inhibition in young adults but were not predictive for reactive inhibition performance. Higher NAA/mIns ratios in the preSMA and RIFG and lower mIns levels in the OCC were associated with better deployment of proactive and reactive inhibition in older adults. Overall, these findings suggest that altered regional concentrations of NAA and mIns constitute potential biomarkers of suboptimal inhibitory control in aging.

## Introduction

Inhibition plays a critical role in the control of many cognitive and motor functions (Logan et al. 1984; Garavan et al. 1999; Coxon et al. 2006; Aron 2007; Coxon et al. 2012; Fujiyama, Hinder, Schmidt, Garry, et al. 2012a, Fujiyama, Hinder, Schmidt, Tandonnet, et al. 2012b; Coxon et al. 2016; Hermans et al. 2018; Hermans et al. 2019; Cuypers et al. 2020; for reviews see Levin et al. 2014; Tan et al. 2019). As viewed from a motor control perspective, motor inhibition is required during withdrawing, reprogramming, termination, or selection of voluntary movements (Miyake et al. 2000; Stinear et al. 2009; Coxon et al. 2010; Coxon et al. 2012; Mirabella 2014; Bender et al. 2016; Hermans et al. 2018; for a review see: Tan et al. 2019). More generally, it is conceptualized as a process that limits the spreading of neural activity to or from nearby regions that are not relevant to the task at hand (Talelli et al. 2008; Levin et al. 2014) or that downregulates attentional processes directed toward irrelevant stimuli, thus keeping attention focused sharply on the task (Diamond 2013; Hsieh et al. 2016). Motor inhibitory processes can be classified as reactive (i.e., cessation of a motor response that is already in progress) or proactive (i.e., inhibitory control mechanisms engaged prior to the initiation of a response) (Logan et al. 1984; Aron 2011; Leunissen et al. 2016; Meyer and Bucci 2016). Reactive inhibition can be viewed as an abrupt stopping of an already planned or initiated action in response to an external cue (Logan et al. 1984; Aron 2011). In contrast, proactive inhibition is expected to be more dominant when a potential need for action cancelation may require a reactive response (Leunissen et al. 2016; Meyer and Bucci 2016); thus, it is viewed as a process that is generated according to the goals of the subject rather than by an external signal (Aron 2011; Braver 2012).

Reactive and proactive inhibition are thought to be mediated by a network, which includes the right inferior frontal gyrus (RIFG), the pre-supplementary motor area (preSMA), and the basal ganglia (Aron 2011; Leunissen et al. 2016; Zhang and Iwaki 2019). Functional magnetic resonance imaging (fMRI) studies in healthy volunteers have shown that successful reactive inhibition is associated primarily with the activation of a hyperdirect pathway in which the subthalamic nucleus (STN) receives direct inputs from RIFG and preSMA (Nambu et al. 2002; Aron and Poldrack 2006; Aron 2011; Jahfari et al. 2011; Jahanshahi 2013; Coxon et al. 2016; Leunissen et al. 2016; Hell et al. 2018; Zhang and Iwaki 2019; Chen et al. 2020). In contrast, proactive inhibition is thought to rely more heavily on the activation of direct (cortico-striato-nigral) and indirect (cortico-striato-pallido-subthalamo-nigral) pathways (Aron 2011; Jahanshahi 2013; Benis et al. 2014; Leunissen et al. 2016; Zhang and Iwaki 2019). Nonetheless, evidence suggests that the three aforementioned pathways are highly interconnected (e.g., Zhang and Iwaki 2019). Notably, activation of all three pathways involves recruitment of prefrontal subregions which are more prone to age-related structural changes than posterior areas (Pfefferbaum et al. 2005; Bonifazi et al. 2018). However, evidence indicates that normal aging is characterized by a progressive decline of reactive inhibition, whereas proactive inhibition appears to remain intact (Coxon et al. 2012; Smittenaar et al. 2015; Bloemendaal et al. 2016; Coxon et al. 2016; Kleerekooper et al. 2016).

Considering that neurochemical alterations could be indicative of neurodegenerative processes at the neuronal network levels, one would expect to find associations between age-related differences in the regional levels of neurochemicals and/or neurotransmitters and reduced inhibitory control in an apparently healthy older population. Indeed, a recent study using ^1^H-MRS (proton magnetic resonance spectroscopy) showed that lower levels of gamma-aminobutyric acid (GABA) in the preSMA were linked to deficient regulation of reactive inhibition in older adults (Hermans et al. 2018). Furthermore, MRS-assessed levels of glutamate–glutamine complex (Glx), *N*-acetylaspartate (NAA), and myo-inositol (mIns) were found to be strong predictors of motor performance declines in older adults (Zahr et al. 2013; Levin et al. 2019). However, only very few attempts have been made so far to study the neurometabolic correlates of reactive or proactive inhibitory processes in “normal” aging (Lorenz et al. 2015; Hermans et al. 2018).

Here, we aim to bridge this gap in knowledge by examining age-related differences in integrity of the neurochemical systems in cortical and subcortical brain regions that are thought to be involved in the mediation of reactive and proactive inhibition. ^1^H-MRS was used for in vivo quantification of brain neurometabolites in the RIFG, preSMA, bilateral sensorimotor cortices (SM1), bilateral striatum (STR), and occipital cortex (OCC). Using a stop-signal task (SST), participants were instructed to respond to “go” cues but to withdraw their response if the “go” cue is followed by a “stop” signal (Aron and Poldrack 2006; Coxon et al. 2007; Verbruggen and Logan 2009a; Leunissen et al. 2016; Hermans et al. 2018; see review Verbruggen and Logan 2009b). This established task was used to specifically measure the efficiency of the reactive inhibition process by deriving the internal reaction time to the stop signal (i.e., the stop-signal reaction time [SSRT]) as previously described, for example, by Verbruggen and Logan (2009b). It was shown that when the stop-signal probability increases, participants slow down their response on go trials to increase their chance of successfully stopping when a stop signal appears (e.g., Zandbelt et al. 2013). Based on this observation, proactive inhibition efficiency was also quantified by manipulating the probability of an upcoming “stop” and calculating the change in the internal timing response to “go” cues as function of a predetermined likelihood for stopping (Verbruggen and Logan 2009a; Aron 2011; Leunissen et al. 2016).

From a neurochemical perspective, we predicted that lower Glx levels in striatal and/or prefrontal regions would be associated with poorer regulation of both reactive and proactive inhibitory control as both forms of inhibition involve the activation of top-down frontostriatal excitatory projections (Aron 2011; Zhang and Iwaki 2019). This hypothesis was inspired by findings from repetitive tanscranial magnetic stimulation (rTMS) studies, showing that excitatory rTMS to the preSMA and inhibitory rTMS to the RIFG significantly improved SST performance, whereas inhibitory rTMS to the preSMA significantly impaired SST performance (Zandbelt et al. 2013; Watanabe et al. 2015; see review Tan et al. 2019). We also predicted that deficient reactive inhibition in older adults would be associated with decreased regional levels of NAA and increased regional levels of mIns across multiple nodes of the prefrontal-basal-ganglia pathways. Decreased regional levels of NAA and increased regional levels of mIns are generally considered to be biomarkers of white matter (WM) microstructural declines and demyelination (Wijtenburg et al. 2013; Grossman et al. 2015). Elevated mIns and reduced NAA (and overall lower NAA/mIns ratio) are considered as robust markers of neurodegenerative processes, reflecting the combined pathology of decreased neuronal integrity and gliosis. For example, lower NAA/mIns ratio in the posterior cingulate cortex of cognitively normal older adults was found to be related to a risk of developing clinical Alzheimer’s disease (Waragai et al. 2017). Finally, based on evidence that the preSMA–STN tract connection strength is positively correlated with the efficiency of reactive inhibition in older adults (Coxon et al. 2012, 2016), we expected that poorer reactive inhibition (i.e., longer SSRTs) will be related primarily to lower levels of NAA and elevated levels of mIns in the preSMA.

## Materials and Methods

### Participants

We included 30 healthy young adults (14 men; mean age ± standard deviation [SD], 23.2 ± 4.3 years; age range, 18.3–33.8 years) and 29 healthy older adults (13 men; mean age ± SD, 67.5 ± 3.9 years; age range, 60.2–73.8 years) that were from the same sample as in Hermans and colleagues (Hermans et al. 2018). All participants were right-handed (Oldfield 1971), had no past or present history of neurological or psychiatric disorders, had no contraindications for magnetic resonance imaging (as indicated in the guidelines of the University Hospital Leuven), had normal or corrected to normal vision, and reported no consumption of psychoactive medications at the time of the experiment. The experimental protocol was approved by the local Medical Ethics Committee for Biomedical Research (University Hospital Leuven; approval number s58333), and a written informed consent was obtained from all participants prior to their inclusion in the study.

### Stop-Signal Task (SST)

**Figure 1:**
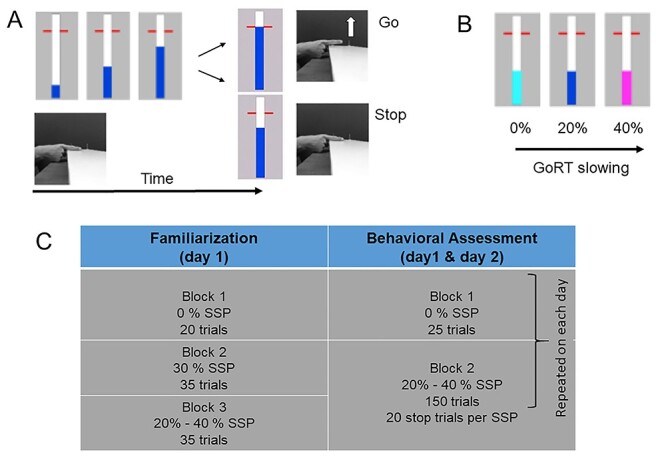
Behavioral task and experimental protocol. (*A*) Stop-signal task: participants had to rest their right index finger on a switch (see also Hermans et al. 2018). On a computer screen, a bar started to fill at a constant and equal rate, crossing a horizontal target line at 800 ms. In “go” trials, participants had to stop the indicator as close as possible to the red target. In “stop” trials, the bar would stop filling before it reached the target line. Participants had to cancel the movement of lifting their finger/releasing the switch. (*B*) The color of the bar was light blue, dark blue, or magenta for the 0%, 20% and 40% stop-signal probability, respectively. (*C*) Experimental protocol consisted of familiarization session (day 1) and two behavioral assessment sessions on separate days, with at least 48 h in between sessions (see text for details).

**Figure 2:**
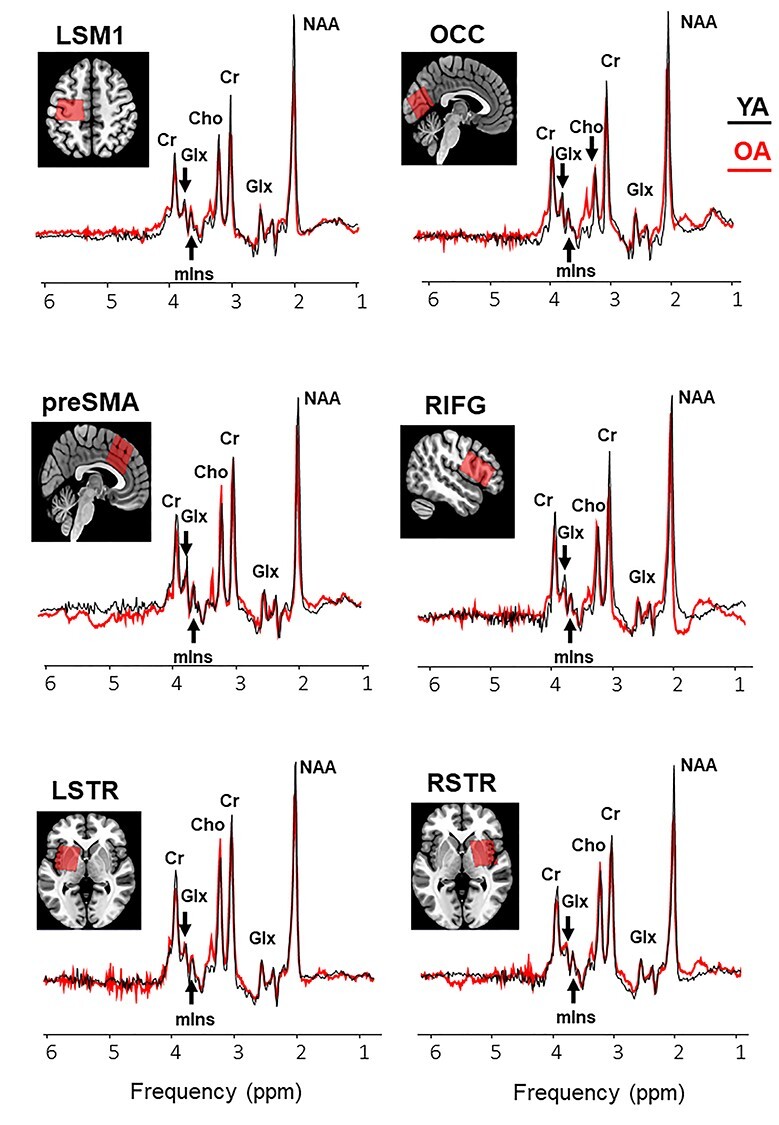
Example voxel positions (coregistered to T1) and group average spectra from young (black curve) and older (red curve) participants (right sensory motor cortex is not shown). Group means and variability measures of neurometabolite levels and tissue segmentation characteristics in the tested voxels are reported in Supplementary Table 1. Abbreviations: NAA, *N*-acetylaspartate; Glx, glutamate–glutamine complex; mIns, myo-inositol; Cho, choline; Cr, creatine + phosphocreatine; LSM1, left sensorimotor cortex; LSTR, left striatum; OCC, occipital cortex; preSMA, pre-supplementary motor area; RIFG, right inferior frontal gyrus; RSTR, right striatum.

The SST and corresponding data have been previously reported (see Hermans et al. 2018). Briefly, participants performed an anticipated response version of the SST (Leunissen et al. 2016; Hermans et al. 2018; Hermans et al. 2019). A vertical indicator (Fig. 1*A*, blue bar) was shown on a computer screen (refresh rate, 60 Hz), which moved upward at a constant speed on each trial, crossing a horizontal target line at 800 ms from the onset. Participants were instructed to stop the indicator as close as possible to the target line by lifting the right index finger from the switch (operating force, 0.10 N; catalog #V-7-2B17D8-162, Honeywell). The color of the target line changed immediately after the completion of each go trial according to the response time (green, yellow, orange, or red for responses within 20, 40, 60, or more than 60 ms difference from the target line, respectively) to provide feedback to the participants on their performance. In some trials, the indicator stopped before reaching the target line (i.e., stop trial), and participants were instructed to cancel the planned finger lift. A dynamic staircase algorithm was used to adjust the time that the indicator stopped (i.e., stop-signal delay [SSD]) by increasing (after a successful stop trial) or decreasing (after an unsuccessful stop trial) the SSD in steps of 33 ms in order to obtain a near-to-equal numbers of successful and unsuccessful stop trials (i.e., probability to inhibit—*P*(inhibit) ~ 50%). To assess proactive inhibition, three stop-signal probability (SSP) conditions (0%, 20%, and 40%) were presented in a blocked order. The color of the indicator was set at light blue for a 0% SSP condition (i.e., only go trials), dark blue for the 20% SSP condition, and magenta for the 40% SSP condition (Fig. 2*B*). Participants were instructed to perform the task as accurately as possible (aim for green or yellow lines after “go” trials). They were informed that it would not be possible to cancel the movement of lifting their finger on all stop trials. The participants were told that no stops would occur when the indicator was light blue and that the probability of stops was higher when the indicator was magenta compared with dark blue.

Participants completed two behavioral assessment sessions that were conducted on two separate days with at least 48 h in between sessions (Fig. 1*C*). On the first day, participants performed three practice blocks (familiarization session): one with 0% SSP (20 trials), one with 30% SSP (35 trials), and one with both 20% and 40% SSP (35 trials), respectively. After the practice blocks, participants were asked to describe the difference between the colors to make sure that the instructions were understood correctly. Next, they completed the first behavioral assessment session. Here, the 0% SSP condition was presented in the first block, which consisted of 25 trials. The 20% and 40% SSP conditions were randomly presented within the second block, which consisted of 150 trials with a matching number of “stop” trials across the conditions (80 “go” trials and 20 “stop” trials for the 20% SSP condition; 30 “go” trials and 20 “stop” trials for the 40% SSP condition). The inter-trial interval was set to 3.25 s, and the indicator was set to empty 1.28 s after trial onset in each block. The same testing procedure was repeated on the second day (second behavioral assessment session), resulting in a total of 350 test trials. Four young adults did not complete the behavioral assessment sessions.

### Behavioral Data Analysis

Behavioral data analysis was performed for the two behavioral assessment sessions separately, and the results were averaged across sessions. Primary performance measures of the SST were the mean “go” task response time for 0% SSP (GoRT) (i.e., the internal timing response to the “go” cue on trials with 0% probability for a stop signal), SSRT (a measure of reactive inhibition), and GoRT_40–20_ (a measure of proactive inhibition). The response time on go trials (GoRT) was measured as the time between the start of the trial and the moment when the finger was lifted from the switch. A GoRT of 800 ms reflects a perfectly timed response. go trials with early response times (<400 ms) or no response (>1280 ms) were removed (Hermans et al. 2018). SSRT was calculated across the 20% and 40% SSP conditions with the integration method (Logan et al. 1984; Verbruggen and Logan 2009b). Specifically, “go” RTs were rank ordered, and SSRT was obtained by subtracting the mean SSD from the nth “go” RT, where *n* is obtained by multiplying the number of “go” RTs by *P*(inhibit) (Logan et al. 1984). Longer SSRTs are indicative of less-efficient reactive inhibitory control. Finally, proactive inhibition was calculated as the difference in “go” RTs between trials in the 40% SSP and 20% SSP condition (i.e., GoRT_40–20_ = GoRT_40_ - GoRT_20_), representing the amount of proactive slowing (GoRT_40_ and GoRT_20_ are the “go” response times measured at the 40% SSP and 20% SSP conditions, respectively). Secondary performance measures were SSD, response time on failed stop trials (RT_SF_), and *P*(inhibit). The primary and secondary performance measures (group Means ± SD) of the stop-signal task (SST) are summarized in Table 1 for young (*n* = 25) and older (*n* = 26) adults who had a complete dataset of MRI and performance measures.

**Table 1:** Summary of performance measures (group means ± SD) on the stop-signal task (SST) in young (YA) and older (OA) adults

		YA (*n* = 25)	OA (*n* = 26)	*t*(49)	*P*
Primary measures					
GoRT (ms)^a^		815.5 (9.5)	821.6 (20.6)	-1.333	0.1888
GoRT_40–20_ (ms)	Proactive inhibition^b^	10.26 (6.14)	13.01 (16.77)	-0.770	0.4450
SSRT (ms)	Reactive inhibition^c^	**193.1 (12.5)**	**206.1 (18.0)**	**−2.989**	**0.0044**
Secondary measures					
RT_SF_ (ms)		796.7 (10.0)	785.4 (26.6)	1.987	0.0525
SSD (ms)		616.5 (13.9)	608.4 (24.5)	1.442	0.1557
*P*(inhibit) (%)		52.60 (1.02)	52.50 (1.12)	0.334	0.7398

Notes: GoRT, go response time in SST trials with no stop signal (0% SSP); GoRT_40–20_, difference in Go RTs between SST trials in the 40% and 20% stop-signal probability (SSP) conditions; SSRT, stop-signal reaction time; RT_SF_, response time on failed stop trials; SSD, stop-signal delay. Performance measures showing significant group differences (*P* values < 0.05) are indicated in bold. All included participants had a complete dataset of MRI and performance measures.

^a^Target GoRT = 800 ms.

^b^Higher values represent better proactive inhibition.

^c^Higher values represent poorer reactive inhibition.

### MRI Acquisition

Scanning was performed on a Philips 3 T Achieva Dstream System (Philips Healthcare) equipped with a 32-channel receiver head coil (Hermans et al. 2018). The imaging protocol consisted of a high-resolution 3D T1-weighted structural image (3D turbo field echo (TFE); repetition time (TR) = 9.6 ms; echo time (TE) = 4.6 ms; resolution = 0.98 × 0.98 × 1.2 mm^3^; 185 coronal slices) in the first session and two short 3D T1-weighted structural images (3D TFE; TR = 9.6 ms; TE = 4.6 ms; resolution = 1.2 × 1.2 × 2 mm^3^; 111 coronal slices) in subsequent imaging sessions to verify voxel positioning for MRS. MRS data were acquired using the MEGA-PRESS spectral editing method (Mescher et al. 1998) with the following acquisition parameters: 14-ms editing pulses at 7.46 ppm (edit-OFF) and 1.9 ppm (edit-ON); TE = 68 ms; TR = 2 s; 320 averages; 2-kHz spectral width; and MOIST (multiple optimizations insensitive suppression train) water suppression, resulting in a total acquisition time of 11 min. Unsuppressed water signals (PRESS) were acquired from all volumes of interest for absolute metabolite quantification in an interleaved manner (Hermans et al. 2018), using the same acquisition parameters, except for number of averages = 16. GABA-edited MRS findings were reported in a previous study (Hermans et al. 2018) and will not be reported here.

Volumes of interest (VOIs) were planned in the bilateral sensorimotor areas (SM1) (left SM1 [LSM1] and right SM1 [RSM1]; both voxel size: 3 × 3 × 3 cm^3^), bilateral preSMA (voxel size: 3 × 3 × 3 cm^3^), right inferior frontal cortex RIFC (voxel size: 4 × 2.5 × 2.5 cm^3^), bilateral striatum (STR) (left STR [LSTR] and right STR [RSTR]; both voxel size, 3 × 3 × 3 cm^3^), and the bilateral occipital cortex (OCC) (voxel size: 3 × 3 × 3 cm^3^) (Hermans et al. 2018). The aforementioned subregions were selected on the basis of their functional relevance to inhibitory control of movements, as shown from previous fMRI studies (e.g., Aron and Poldrack 2006; Watanabe et al. 2015; Coxon et al. 2016; Leunissen et al. 2016); see reviews (Aron 2011; Tan et al. 2019). The imaging protocol started with the long high-resolution T1 scan followed by three MRS scans. After a short break outside the scanner, a short (low-resolution) T1 scan was acquired followed by two MRS scans, a short T1 scan, and an MRS scan. All regions were acquired in a random order, except that the LSTR was followed by the RSTR or vice versa. The T1-weighted MR images were used to position the voxels according to anatomical landmarks (Fig. 2, representative voxel positions). The LSM1/RSM1 voxels were centered over the left/right-hand knob (Yousry et al. 1997) parallel to the anterior–posterior axis with one surface parallel to the cortical surface in the coronal and axial views (Greenhouse et al. 2016). For the preSMA voxel, a horizontal line was drawn between the anterior commissure (AC) and the posterior commissure in the sagittal plane, and a perpendicular line was constructed to this line through the AC. The preSMA voxel was centered over the median line with the posterior superior corner intersecting the perpendicular line (Behrens et al. 2006; Kim et al. 2010). Subsequently, it was aligned with the cortical surface in the sagittal view. The RIFG voxel was positioned above the temporal lobe and centered over the inferior frontal gyrus, with the longest axis extending anterior to posterior, parallel to the cortical surface. The striatal (LSTR and RSTR) voxels were centered over the putamen. In the coronal and axial view, we checked that the voxel was not positioned in the ventricle, and, as a consequence, only part of the caudate was covered. The OCC voxel was centered on the median line, positioned as posterior as possible and aligned with the cerebellar tentorium in the sagittal plane (Puts et al. 2011).

### MRS Analysis

All metabolites were quantified from the OFF spectra (e.g., Maddock et al. 2018). In total 210 spectra (= 7 VOIs × 30 participants) from young adults and 203 spectra (= 7 VOIs × 29 participants) from older adults were processed. Metabolite signals and corresponding non-suppressed water signals were quantified using the QUEST (quantitation based on quantum estimation) module in jMRUI v6.0 (Stefan et al. 2009; Weerasekera et al. 2019). Signal-to-noise ratios (SNR) were determined by jMRUI QUEST in the time domain (maximum of FID/standard deviation of FID tail). Only spectra with linewidths less than 10 Hz and SNR greater than 5 were included for quantification. Spectra were also visually assessed to ensure the absence of artifacts. A total of 16 spectra were excluded due to low data quality (two spectra from one young adult and 14 spectra from three older adults). The excluded data were eliminated from further processing. Water-referenced concentrations of NAA, Glx, Cr (creatine + phosphocreatine), Cho, and mIns were quantified for each of the seven voxel locations. Averaged spectra from young and old participants are shown in Figure 2. The MPRAGE T1-weighted MR images, acquired for the localization and placement of the MRS voxels, were segmented with a statistical parametric mapping approach using SPM8 (http://www.fil.ion.ucl.ac.uk/spm/). Voxel registration was performed using custom-made scripts developed in MATLAB (MathWorks, Natick, Massachusetts, USA), which can be accessed at http://biu.bangor.ac.uk/projects.php.en (Sanaei Nezhad et al. 2017). Using the T1-weighted MR image and the orientation and location information from the Philips SPAR files, the scripts generated a binary mask of the voxel location. After creating a binary mask on the native T1-weighted MR image, SPM8 was used to segment the T1 image into gray matter (GM), WM, and cerebrospinal fluid (CSF) and calculate the respective partial volume fractions within the binary mask. SPM uses a diffeomorphic algorithm to warp individual subject images into MNI space and generate spatially normalized and smoothed Jacobian scaled images, thereby normalizing the WM and GM sensitivities in the T1 images. The application of this procedure enables the calculation of percentages of each tissue type within each of the seven VOIs. The partial volumes (expressed in percentage) of GM, WM, and CSF are summarized in Supplementary Table 1. The individually segmented tissue fractions were then used to correct for metabolite concentrations quantified using QUEST for differences in CSF content according to Gasparovic and colleagues (Gasparovic et al. 2006). Metabolite T1 and T2 relaxation times that were used for calculating the final corrected metabolite concentrations in the present study were taken from existing literature (Wansapura et al. 1999; Träber et al. 2004). The used T1 values were 1331 ms for GM, 832 ms for WM, and 3817 ms for CSF. The used T2 values were 110 ms (GM), 79 ms (WM), and 503 ms (CSF).

### Statistical Analysis

Eight of 59 participants were excluded from the final analysis due to missing data (four young adults) and/or poor quality of MRI/MRS acquisition (one young adult and three older adults). Group differences in neurometabolite concentrations (NAA, Glx, total Cr, Cho, and mIns) and the NAA/mIns concentration ratio were evaluated with a series of Student’s *t*-tests with age group as independent variable. A false discovery rate (FDR) controlling procedure for multiple comparisons to reduce probability of type 1 error was used with a 0.05 significance level. The critical FDR *P* value was <0.021 (see Supplementary Table 1). Bivariate correlation analyses (Pearson’s *r*) were performed to investigate the relationship between the selected neurometabolic measures (i.e., tissue-corrected Glx, NAA, Cho, and mIns levels, and NAA/mIns ratios) in all seven regions of interest (ROIs) and the three selected (principal) performance measures of the SST (i.e., GoRT, GoRT_40–20_, and SSRT). Correlation analyses were performed separately for the young and older adults. Based on the results of the correlation analyses, multiple regression analyses were performed to determine the unique variance contributed by the aforementioned neurometabolic measures to the “go” response time for 0% SSP (i.e., GoRT in the trials with no stop signal), proactive inhibition (i.e., GoRT_40–20_), and reactive inhibition (i.e., SSRT). Putative (candidate) neurometabolic measures in each ROI were selected based on the results of the correlation tests and were entered into the regression model if they were correlated with the performance measure at a significance level of *P* < 0.05 (e.g., Levin et al. 2019). Regression analyses were performed separately on the performance measures and selected neurometabolites from each age group. Full correlation matrixes are presented in Supplementary Table 3 (for GoRT), Supplementary Table 4 (for GoRT_40–20_), and Supplementary Table 5 (for SSRT). Results of the multiple regression models with the main putative neurometabolite predictors of performance are summarized in Table 3 (for the results of the multiple regression models with the set of the putative neurometabolite predictors selected based on the findings of the correlation analyses, see Supplementary Table 6).

**Figure 3:**
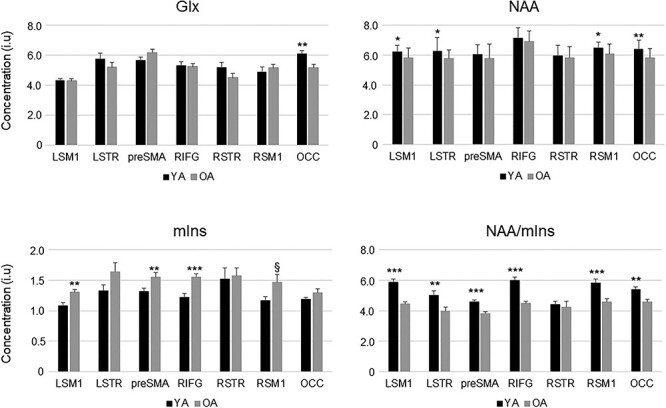
Tissue-corrected NAA, Glx, and mIns levels and NAA/mIns ratio in young adults (black bars) and older adults (gray bars). Bar plot shows mean values; error bars show standard error of mean (SEM). Abbreviations: NAA, *N*-acetylaspartate; Glx, glutamate–glutamine complex; mIns, myo-inositol; LSM1, left sensorimotor cortex; LSTR, left striatum; preSMA, pre-supplementary motor area; RIFG, right inferior frontal gyrus; RSTR, right striatum; RSM1, right sensorimotor cortex; OCC, occipital cortex. Significant group differences are indicated: *P*-levels: ^*^P < *P*(FDR); ^*^^*^P < 0.01; ^*^^*^^*^P < 0.001; *P*(FDR) ≤ §*P* < 0.05. FDR = false discovery rate [*P*(FDR) = 0.021].

## Results

### Performance Measures and Age

Performance data were similar to that reported in a previously published work using the same task and sample (Hermans et al. 2018), showing that only reactive inhibition efficiency was compromised as function of age. Specifically, older adults had significantly longer SSRTs compared with young adults (*t*(49) = −2.989, *P* = 0.004). There was no significant effect of age on the remaining performance measures (all |*t*(49)| ≤ 1.987, *P* ≥ 0.052); for details see Table 1. Finally, there were no significant associations between the three performance measures (i.e., GoRT, GoRT_40–20_, and SSRT) and individual differences in GM and WM tissue fractions; both age groups and all ROIs: |*r*| ≤ 0.348, *P*s ≥ 0.082.

### MRS Measures and Age

Group means of Glx, NAA, and mIns levels and NAA/mIns ratios for the seven ROIs are illustrated in Figure 3. As compared with young adults, older adults showed significantly lower NAA/mIns ratios (in all ROIs except the RSTR), higher mIns levels (LSM1, RIFG, and preSMA), and lower NAA levels (LSM1 and OCC); all *P* ≤ 0.0197. In addition, we found significant elevations of Cho levels as function of age in five of seven ROIs, including the bilateral SM1, bilateral striatum, and RIFG (data not shown in the figure); all *P* ≤ 0.0187. Glx and total Cr levels were overall stable as a function of age (in all ROIs except the OCC for Glx); all *P* ≥ 0.0348 (see Supplementary Table 1A for details). The decreased levels of NAA, increased levels of mIns, and the overall reduction of NAA/mIns ratio with age suggest that older individuals exhibited some degree of neurodegenerative change in most ROIs. The question emerges whether the observed group differences in neurometabolite levels are associated with age-related changes in tissue composition, which were marked by significant or trend-level declines of GM volume with age in all seven VOIs (Supplementary Table 1B; see also in Hermans et al. 2018). However, significant associations between NAA, Glx, Cr, Cho, and mIns levels or NAA/mIns ratio that were not corrected for tissue volume fractions and fractional tissue volumes of GM and WM were observed mainly in young adults (Supplementary Table 2), suggesting that the observed effect of age on neurometabolite levels cannot be explained primarily by individual differences in tissue fractions.

### MRS Correlates of Behavioral Performance

#### MRS Correlates of GoRT

**Figure 4:**
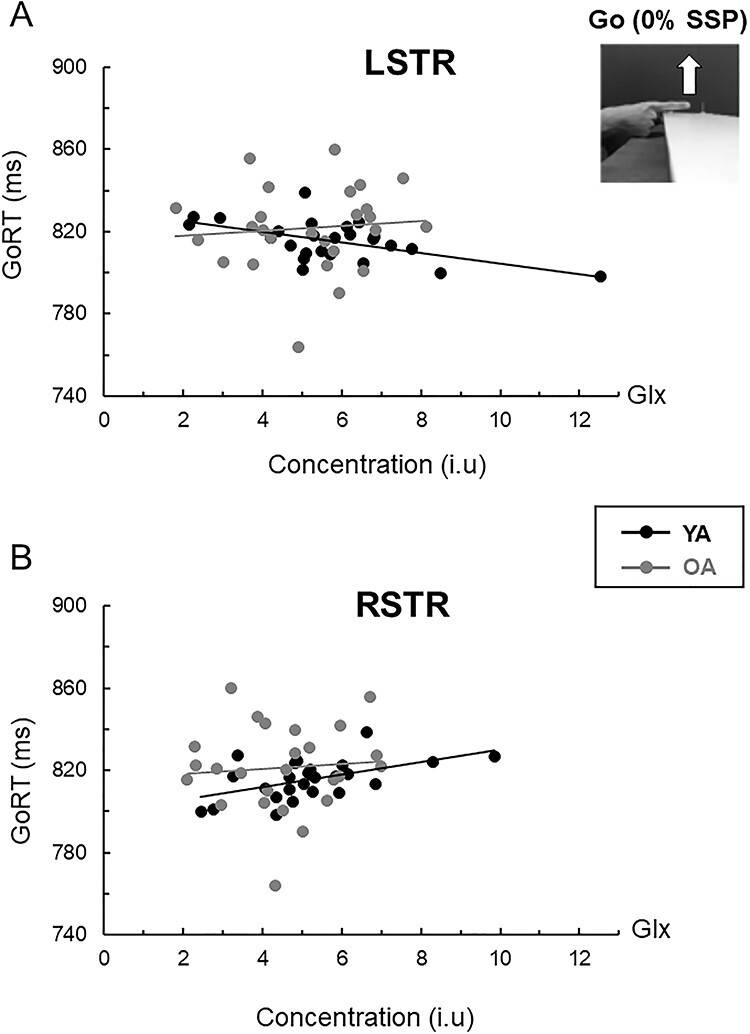
Relationship between tissue-corrected striatal Glx and response time on go trials (GoRT) in young (black circles/lines; *n* = 25) and older (gray circles/lines; *n* = 26) adults. For young adults: (*A*) Higher Glx level in the left striatum (LSTR) predicted shorter response time (*r* = −0.572, *P* = 0.003). (*B*) Higher Glx level in the right striatum (RSTR) predicted longer response time in young adults (*r* = 0.521, *P* = 0.008). No significant associations between striatal Glx levels and GoRT were found in older adults (both: |*r*| < 0.1).

For young adults, shorter GoRTs (i.e., early response times at 0% SSP) were associated primarily with higher Glx (*r* = −0.572*, P* = 0.003) levels in the LSTR, whereas longer GoRTs (i.e., late response times at 0% SSP) were associated with higher Glx levels in the RSTR (*r* = 0.521, *P* = 0.008) (Fig. 4). Other putative predictors were LSTR Cho (*r* = −0.418), LSTR mIns (*r* = −0.410), LSTR NAA (*r* = −0.468), and RSM1 NAA (*r* = 0.412); all *P*s ≤ 0.042 (Table 2); a full correlation matrix is presented in Supplementary Table 3. The multiple regression model including the aforementioned six neurometabolites indicated that left striatal NAA, Cho, and mIns contributed only for 3.2% of the variance of GoRT (Supplementary Table 6). The three remaining metabolites (i.e., LSTR Glx, RSTR Glx, and RSM1 NAA) contributed 58.0% of the variance in the GoRT and were significantly associated with change in this measure (all *P*s < 0.05). Parsing the variance indicated that LSTR Glx (16.2%, *P* = 0.0097) was the most prominent predictor followed by RSTR Glx (11.1%, *P* = 0.0284) and RSM1 NAA (12.8%, *P* = 0.194) which accounted for nearly similar portions of the variance considered (Table 3).

**Table 2:** Significant correlations between neurometabolite levels (or ratios) at different brain locations and performance measures on the stop-signal task (SST) in young (YA) and older (OA) adults^a^

		GoRT		GoRT_40–20_		SSRT	
Metabolite	Location	YA (*N* = 25)	OA (*N* = 26)	YA (*N* = 25)	OA (*N* = 26)	YA (*N* = 25)	OA (*N* = 26)
Glx	LSTR	−0.572^**^	—	—	—	—	—
	RSTR	0.521^**^	—	0.397^*^	—	—	—
NAA	LSM1	—	0.510^**^	—	—	—	—
	LSTR	−0.468^*^	0.480^*^	—	—	—	—
	RSM1	0.421^*^	0.412^*^	—	—	—	0.457^*^
mIns	LSTR	−0.410^*^	—	—	—	—	
	OCC	—	—	—	—	—	0.584^**^
	RSM1	—	0.430^*^	—	—	—	—
Cho	LSRT	−0.418^*^	—	—	—	—	—
	RSM1	—	—	—	—	—	0.470^*^
NAA/mIns	OCC	—	—	—	—	—	−0.465^*^
	preSMA	—	—	—	—	−0.474^*^	—
	RIFG	—	—	—	0.382^‡^	—	—

Notes: ^a^Full correlation matrixes are presented in Supplementary Table 3 (GoRT), Supplementary Table 4 (GoRT_40–20_), and Supplementary Table 5 (SSRT).

^^*^^Significant correlations (Pearson’s *r*) at *P* < 0.05 (uncorrected).

^^*^^*^^Significant correlations (Pearson’s *r*) at *P* < 0.01 (uncorrected).

^‡^Marginal effect (uncorrected *P* = 0.054).

Abbreviations: LSM1, left sensorimotor cortex; LSTR, left striatum; OCC, occipital cortex; preSMA, pre-supplementary motor area; RIFG, right inferior frontal gyrus; RSTR, right striatum; RSM1, right sensorimotor cortex; Glx, glutamate–glutamine complex; NAA, *N*-acetylaspartate; mIns, myo-inositol; Cho, choline; NAA/mIns, NAA to mIns ratio.

**Table 3:** Multiple linear regression model summary for the principal neurometabolite predictors of GoRT (young and old) and SSRT (old)

Performance/Age group	*R* ^2^	*R* ^2^-Adj.	*F*	Contributing neurometabolite	*R* ^2^ change^‡^	β (SE)	*B* (SE)	*P* value
GoRT YA	0.580	0.520	9.657^^*^^*^^					
				Intercept			760.3 (23.10)	0.0000
				**LSTR [Glx] ↓**	0.162	−0.427 (0.150)	−1.954 (0.686)	**0.0097**
				**RSTR [Glx] ↑**	0.111	0.353 (0.150)	2.078 (0.883)	**0.0284**
				**RSM1 [NAA] ↑**	0.128	0.360 (0.142)	8.621 (3.407)	**0.0194**
GoRT OA	0.399	0.317	4.868^^*^^*^^					
				Intercept			702.9 (36.57)	0.0000
				LSM1 [NAA]	0.072	0.306 (0.188)	8.809 (5.421)	0.1184
				LSTR [NAA]	0.066	0.286 (0.184)	9.687 (6.227)	0.1340
				RSM1 [mIns]	0.053	0.247 (0.178)	7.783 (5.614)	0.1795
SSRT OA	0.463	0.416	9.898^^*^^*^^*^^					
				Intercept			105.6 (26.93)	0.0007
				**OCC [mIns] ↑**	0.254	0.514 (0.156)	30.97 (9.397)	**0.0032**
				**RSM1 [NAA] ↑**	0.122	0.356 (0.156)	9.909 (4.336)	**0.0318**

Notes: Directions of associations between performance measures and models’ principal neurometabolite predictors are indicated: (↑) for positive association and (↓) for negative association; significant associations (*P* values < 0.05) are indicated in bold. Multiple regression model *R*^2^, Adjusted *R*^2^ (*R*^2^-Adj), and *F*-value (*P* levels: ^^*^^P < 0.05; ^^*^^*^^P < 0.01; ^^*^^*^^*^^P < 0.001); *B*, regression coefficient; β, standardized regression coefficient; SE, standard error. Abbreviations: NAA, *N*-acetylaspartate; Glx, glutamate–glutamine complex; mIns, myo-inositol; RSM1, right sensorimotor cortex; RSTR, right striatum; LSM1, left sensorimotor cortex; LSTR, left striatum; OCC, occipital cortex.

^‡^ *R* ^2^ change represent the amount by which *R*^2^ is reduced if a particular independent variable is removed from the model.

For older adults, significant positive associations were observed between longer GoRT and NAA levels in the LSM1 (*r* = 0.510, *P* = 0.008), LSTR (*r* = 0.480, *P* = 0.013), and RSM1 (*r* = 0.412, *P* = 0.037) and mIns levels in the RSM1 (*r* = 0.430, *P* = 0.028). Together, the four region-specific metabolites contributed to 39.9% of the variance in the GoRT. However, none was a significant GoRT predictor (all, *P*s < 0.1). Parsing the variance indicated that LSTR NAA and LSM1 NAA together accounted for 13.8% of the variance (Table 3), suggesting that longer response times at 0% SSP were associated, primarily, with higher NAA levels in these two regions. Interestingly, the observation that LSTR NAA was positively correlated with longer GoRT in older adults (*r* = 0.480) was opposite to the direction of the association between these variables in young adults where a negative association between LSTR NAA and GoRT was found (*r* = −0.486; Fisher *r*-to-*z* transformation: *z* = −3.53, *P* < 0.001).

#### MRS Correlates of Proactive Inhibition (GoRT_40–20_)

**Figure 5:**
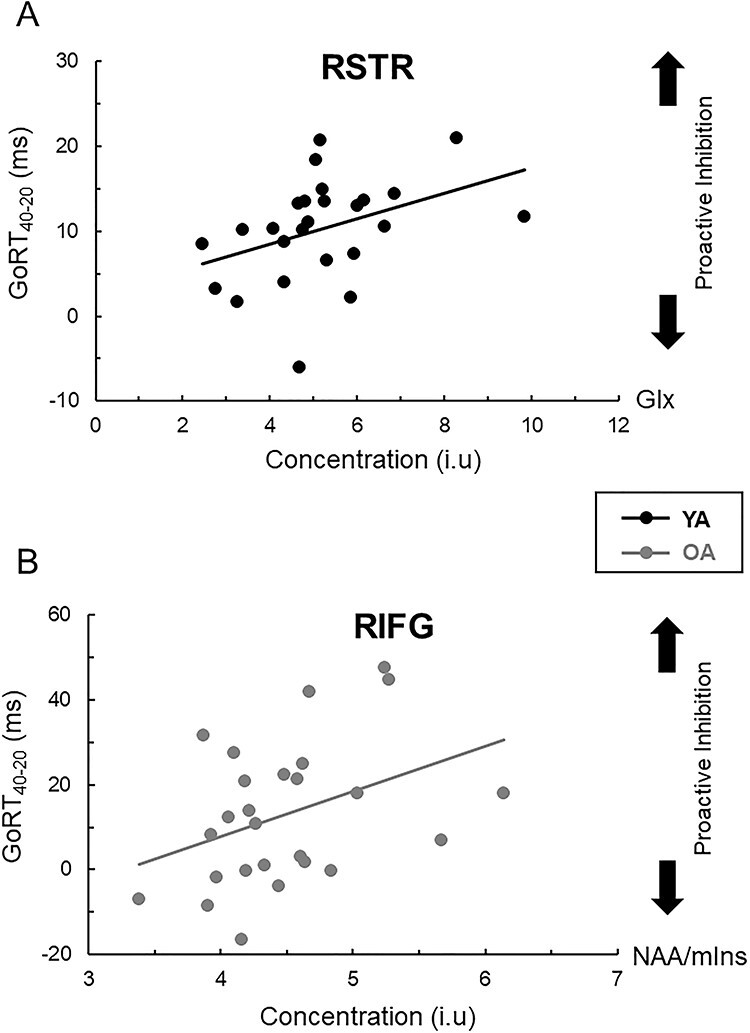
(*A*) Association between GoRT_40–20_, a measure of proactive inhibition efficiency, and tissue-corrected Glx levels in the right striatum (RSTR) in young adults (*r* = 0.397, *n* = 25, *P* = 0.049). (*B*) Association between GoRT_40–20_ and NAA/mIns ratio in the inferior frontal gyrus (RIFG) in older adults (*r* = 0.382, *n* = 26, *P* = 0.054). Longer GoRT_40–20_ measures are indicative of more-efficient proactive inhibitory control.

For both age groups, associations between GoRT_40–20_ and the MRS measures were either marginally significant or did not reach significance (all *P*s ≥ 0.049) (Table 2); a full correlation matrix is presented in Supplementary Table 4. Based on the trends shown, a higher degree of proactive inhibition (expressed by positive/longer GoRT_40–20_) in young adults was weakly correlated with higher levels of Glx in the RSTR (*r* = 0.397, *P* = 0.049), whereas poorer proactive inhibition (expressed by shorter/negative GoRT_40–20_) in older adults was weakly correlated with lower NAA/mIns in the RIFG (*r* = 0.382, *P* = 0.054) (Fig. 5). Overall, these findings suggest that proactive inhibition may rely in part on the integrity of prefrontal-striatal pathways.

#### MRS Correlates of Reactive Inhibition (SSRT)

**Figure 6:**
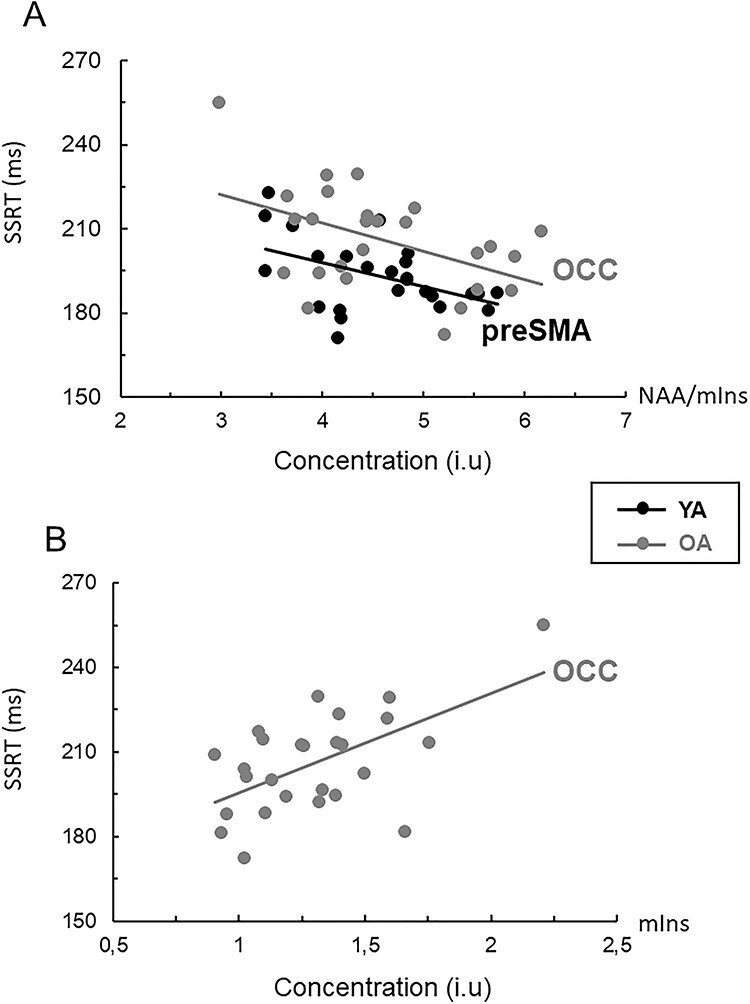
(*A*) Relationship between stop-signal reaction time (SSRT) and NAA/mIns ratio in preSMA (young adults: *r* = −0.474, *n* = 25) and occipital cortex (OCC) (older adults: *r* = −0.465, *n* = 26); both ≤ 0.017. (*B*) Relationship between SSRT and tissue-corrected mIns levels in the OCC (older adults: *r* = 0.584, *n* = 26, *P* = 0.002). Longer SSRTs are indicative of less-efficient reactive inhibitory control.

Better reactive inhibition (i.e., shorter SSRT) in young adults was associated primarily with higher NAA/mIns ratio in the preSMA (*r* = −0.474, *P* = 0.017), whereas better reactive inhibition in older adults was associated primarily with lower mIns levels in the OCC (*r* = 0.583, *P* = 0.003). In addition we observed a significant negative association between SSRT and OCC NAA/mIns (*r* = − 0.464, *P* = 0.017) and significant positive associations between SSRT and SM1 NAA (*r* = 0.457, *P* = 0.019) and SM1 Cho (*r* = 0.470, *P* = 0.015) in older adults (Table 2). No significant correlations were found otherwise in either age group (all |*r*| ≤ 0.337, *P* ≥ 0.099); a full correlation matrix is presented in Supplementary Table 5. Next, regression analysis was performed on data obtained from the older adults. Here the multiple regression model indicated that, together, the four neurometabolite measures (i.e., OCC mIns, OCC NAA/mIns, RSM1 NAA and RSM1 Cho) accounted for 46.4% of the variance in SSRT. However, further examination of the findings indicated that the contribution of OCC NAA/mIns and RSM1 Cho was negligible (<1%). A multiple regression model with OCC mIns and SM1 NAA as only predictors of SSRT showed that OCC mIns accounted for 25.4% of the variance in SSRT, whereas RSM1 NAA accounted for 12.2% of the variance (Table 3). Overall, this observation suggests that increased mIns levels in the OCC was related to reduced efficiency of reactive inhibition in older adults, whereas decreased NAA/mIns ratio in the preSMA was associated with reduced efficiency of reactive inhibition in young adults (Fig. 6).

## Discussion

### General Findings

The present study provides novel insights into the neurochemical biomarkers of inhibitory motor control in healthy young and older adults and highlights putative neurometabolic correlates of deficient inhibitory control in normal aging. We primarily focused on subregions of the prefrontal-striatal pathways and visuomotor pathways (i.e., preSMA, RIFG, bilateral striatum, and bilateral sensorimotor cortices), which are functionally associated with age-induced deficits in inhibitory control of movements (e.g., Coxon et al. 2012, 2016; Leunissen et al. 2016; Hermans et al. 2018). Our first main finding suggests that levels of glutamate–glutamine (Glx) in the striatum and preSMA were associated with efficient regulation of proactive inhibition and shorter response times in young adults. However, there was no evidence to indicate that reduced inhibitory (or excitatory) processes in the stop-signal paradigm were associated with decreased preSMA or striatal glutamate–glutamine concentrations in our sample of older adults. Our second main finding suggests that neurochemical properties characterizing structural integrity of neurons within the prefrontal-striatal pathways and the visuomotor pathways (expressed by local levels of NAA, mIns, and/or NAA/mIns ratios in the sensorimotor, striatal, occipital, and/or prefrontal regions) were found to be predictors of reactive inhibition and response time in older adults. As expected, we found age-related alterations in neurometabolite levels across multiple subregions of the prefrontal-striatal pathways and visuomotor pathways, corroborating findings from previous ^1^H-MRS studies of normal aging (Kaiser et al. 2005a, 2005b, Haga et al. 2009; Boumezbeur et al. 2010; Zahr et al. 2013; Ding et al. 2016; Levin et al. 2019; see review Cichocka and Bereś 2018).

### Glx as a Neurochemical Correlate of Striatal Function in Young but Not in Older Adults

Our data revealed possible preliminary indications for a lateralized functional organization of the glutamatergic system in striatum, suggesting that the glutamatergic activation in the left striatum predominantly accelerates the go process, whereas glutamatergic activation in the right striatum proactively slows it down. These findings are consistent with existing literature on the role of cortico-striatal excitatory transmission in movement initiation and inhibition (e.g., Aron 2011). Specifically, we found that higher levels of Glx in the left striatum were related to faster response times on go at 0% SSP, whereas higher levels of Glx in the right striatum were associated with slower response times on go and stronger proactive inhibition (i.e., longer GoRT_40–20_ measures) in young adults. The aforementioned observations are consistent with findings from previous fMRI studies in young adults, showing that “go” and “stop” processes are controlled by different (lateralized) frontostriatal pathways (Aron and Poldrack 2006; Smittenaar et al. 2015; Leunissen et al. 2016). Generally, these fMRI studies showed that the go process (for a right-hand move) was significantly associated with the activation of a contralateral (left) frontostriatal–pallidal pathway, whereas activation of the right striatum during successful versus unsuccessful stopping was positively coupled with elevated brain activation levels in the preSMA, rIFC and STN (e.g., Zandbelt and Vink 2010; Leunissen et al. 2016).

The observation that higher Glx levels in the right striatum were significantly associated with longer GoRT_40–20_ is consistent with the understanding that proactive inhibition is mediated at least in part through activation of glutamatergic neurotransmission in right striatum (Aron and Poldrack 2006; Zandbelt and Vink 2010). This observation is in agreement with the general notion that the right cortico-striato-pallidal pathway plays a greater role when reactive inhibition occurs in the presence of enhanced proactive control (Nambu et al. 2002; Jahfari et al. 2011; Jahanshahi 2013; Zhang and Iwaki 2019). Since reactive inhibition is known to coincide with activation of the STN (e.g., Aron and Poldrack 2006; Zandbelt and Vink 2010; Leunissen et al. 2016), one could speculate based on our findings that glutamatergic signaling within the right striatum may be associated with response slowing rather than the stopping process per se (e.g., Zandbelt and Vink 2010). The aforementioned interpretations should, nonetheless, be made with caution given that Glx reflects the total combined glutamate and glutamine in all tissue within the scanned volume of interest rather than glutamatergic transmission or signaling per se.

We found no overt associations between SSRT measures (reflecting stopping performance) and prefrontal or striatal Glx in either age group. On the one hand, this absence of clear associations is in disagreement with current understanding that reactive inhibition is mediated, predominantly, by glutamatergic projections from preSMA and RIFG into the striatum and STN (Aron and Poldrack 2006; Aron 2007). On the other hand, our findings are consistent with those reported by Lorenz and colleagues (Lorenz et al. 2015) who revealed no significant associations between SSRT and glutamate concentration in the striatum. Nevertheless, the same study clearly demonstrated a positive association between striatal glutamate concentration and BOLD activity in the striatum during response inhibition. Interestingly, findings from a recent study by Hermans and colleagues (2018), using edited MR spectroscopy on the same sample group, showed that longer SSRTs in older adults were associated with lower GABA concentration in the preSMA. Based on this observation, Hermans and colleagues proposed that GABAergic functioning in preSMA may partly contribute to the efficiency of reactive inhibition in older adults (Hermans et al. 2018). The aforementioned observation is complementary to our observations showing that shorter SSRTs in young adults were associated with higher NAA/mIns ratios in the preSMA, supporting the notion that high stopping efficiency may rely in part on superior neurochemical integrity of WM pathways originating in the preSMA, even in young adults. This superior neurochemical integrity of WM is expected to be manifested by higher levels of NAA (e.g., Wijtenburg et al. 2013), lower levels of mIns, and/or higher NAA/mIns ratios as observed in our sample of young adults and elsewhere (see review Cichocka and Bereś 2018).

### NAA and mIns as Neurochemical Correlates of Age-Related Changes in Behavioral Performance

We further examined the assumption that age-related changes in regional levels of NAA and mIns within the nodes of the prefrontal-striatal and visuomotor pathways may have detrimental effects on reactive inhibition. Structural integrity of WM and connection strength within the prefrontal-striatal pathways were found to be principal predictors of individual and age-related differences in the efficiency of reactive inhibition in previous work (Coxon et al. 2012, 2016; Rae et al. 2015). In line with previous studies, we observed a progressive decline in the efficiency of reactive inhibition with age (Coxon et al. 2012, 2016; Smittenaar et al. 2015; Bloemendaal et al. 2016; Kleerekooper et al. 2016). Our current study suggests that decrements in reactive inhibition (i.e., longer SSRTs) in older adults were associated predominantly with increased mIns levels in the occipital cortex (Table 3). Increased mIns levels in normal aging (or in neurological disorders) are thought to be associated with loss of WM microstructural integrity (Wijtenburg et al. 2013; Grossman et al. 2015). Therefore, the observed association between higher OCC mIns and the longer SSRT could suggest a role of neurodegenerative processes within the visual cortex and/or visual-striatal pathways (e.g., Shipp 2017) in reactive inhibition deficits. Together with findings from fMRI and brain stimulation studies (e.g., Coxon et al. 2016; Zhang and Iwaki 2019), our observations suggest that individual differences in the neurochemical integrity of the nodes of the pathways connecting the sensorimotor regions with the right inferior frontal gyrus and preSMA significantly predict individual differences in reactive stopping (Xu et al. 2016; Tsvetanov et al. 2018; see review Tan et al. 2019). Finally, we found that higher levels of NAA in the left striatum were related to faster response times on go at 0% SSP in young adults, but this association was reversed in older adults (Table 2). The aforementioned observation suggest that decreased structural and/or neurochemical integrity within the left striatal pathways may be detrimental for response generation (and possibly internal timing processes associated with this response) in older adults.

Our findings indicate that changes in the levels of mIns and NAA (or NAA/mIns ratios) in the right SM1, left STR, and OCC were significantly related to performance variance on “go” and “stop” in older adults. Increased mIns levels and decreased NAA/mIns ratio have been reported previously in relation to neurodegenerative disorders, which could indirectly signify deterioration in axonal and myelin integrity (Kantarci et al. 2002; Kalra et al. 2006; Ding et al. 2008; Chiappelli et al. 2015; Grossman et al. 2015; Lin et al. 2016; Waragai et al. 2017; Tumati et al. 2018; Weerasekera et al. 2018). Therefore, it is not surprising that within-group differences in the regional levels of NAA and mIns or regional changes in the NAA/mIns ratio (rather than reduced levels of Glx) in older adults largely account for performance differences in SSRT. Additionally, we found trends toward decrements in proactive inhibition (i.e., shorter/negative GoRT_40–20_ values) that were related to decreased NAA/mIns ratios in the preSMA (in young adults) or the right inferior gyrus (in older adults). The aforementioned findings are complementary to data from previous task-related fMRI studies showing that the preSMA, right inferior gyrus, and striatum are also principal nodes of the prefrontal-striatal pathways that underlie proactive behavior (Aron 2007; Rae et al. 2015; Coxon et al. 2016; Leunissen et al. 2016; Zhang and Iwaki 2019).

In spite of this prediction, results of the regression analyses in our study established that neurometabolic alterations in the RIFG and preSMA did not contribute significantly to the variance in the SSRT. But in line with previous findings (Coxon et al. 2012, 2016), one should not exclude the possibility that MRS measures of NAA and mIns levels within WM fiber tracts connecting RIFG, preSMA, and STN may be more relevant for prediction of reactive inhibition compared with NAA and mIns levels in prefrontal and striatal GM. In this respect, future research could further examine the specific contribution of neurometabolite concentrations in GM and WM to performance variance. This could be achieved, for example, through the implementation of whole-brain ^1^H-MR spectroscopic imaging (Gasparovic et al. 2009; Ding et al. 2016) with the aim of identifying overarching parallels between neurometabolite and structural characteristics of the aging brain in order to determine more specifically associations between structural/neurochemical network integrity changes and performance changes.

## Conclusions

We have shown that the processes associated with movement initiation and proactive inhibition depend (at least in part) on the integrity of the glutamatergic systems in the preSMA, the right inferior frontal gyrus, and the striatum. However, our observations suggest that Glx levels were not predictive for reactive inhibition performance in either age group. We further provide some indirect support for a lateralized organization of the frontostriatal pathways pointing to functionally segregated striatal loops for “go” and “stop” behavior. With aging, the ability to effectively regulate these processes appears to become more reliant on the structural and neurochemical integrity of these regions. Based on the present MRS data, we suggest that changes in neurometabolite concentrations associated with structural integrity of the fronto-basal-ganglia pathways, particularly NAA and mIns, are closely associated to motor inhibition declines in normal aging. Specifically, we propose that deficient regulation of motor inhibition can be attributed in part to neurodegenerative processes in WM tracts connecting these regions, which are indirectly characterized by decreased NAA and increased mIns. Future work should examine whether age-associated neurometabolite changes that occur in the WM tracts connecting preSMA, right inferior frontal gyrus, and STN may constitute limiting factors for successful inhibitory control.

## Notes

*Conflict of Interest*: None declared.

## Supplementary Material

Supplemental_Tables_S1-S6_CCC-2020-00058_tgaa028
