# Supplementary material for: Neurometabolic Correlates of Reactive and Proactive Motor Inhibition in Young and Older Adults: Evidence from Multiple Regional 1H-MR Spectroscopy
Source: Cereb Cortex Commun. 2020 Jun 27;1(1):tgaa028. doi: 10.1093/texcom/tgaa028 (PMC8152832; doi:10.1093/texcom/tgaa028)
Supplement: Supplemental_Tables_S1-S6_CCC-2020-00058_tgaa028 [file supplemental_tables_s1-s6_ccc-2020-00058_tgaa028.docx]

**Supplemental Table S1. (A)** Group means, standard deviation (SD), and coefficients of variation (CoV) for the tissue-corrected estimates of N-acetylaspartate (NAA), Glutamate-glutamine complex (Glx), creatine + phosphocreatine (Cr), Choline (Cho), Myo-inositol (mIns), and NAA to mIns ratio (NAA/mIns) in the seven regions of interest (ROIs). **(B)** Group means, SDs, and CoVs for the fractional tissue volumes of gray matter (GM) and white matter (WM) in the seven ROIs.

| **(A)** |  |  |  |  |  |  |  |  |  |  |
| --- | --- | --- | --- | --- | --- | --- | --- | --- | --- | --- |
| ROI/  Neurometabolite | Mean YA | Mean OA | SD  YA | SD  OA | CoV YA | CoV OA | t(49) | p-value | Critical p-value | FDR test |
| RIFG [NAA/mIns] | 6.02 | 4.49 | 1.02 | 0.60 | 17.00 | 13.44 | 6.525 | **0.0000** | 0.0012 | 1 |
| LSM1 [NAA/mIns] | 5.88 | 4.47 | 1.05 | 0.59 | 17.89 | 13.17 | 5.946 | **0.0000** | 0.0024 | 1 |
| preSMA [NAA/mIns] | 4.57 | 3.79 | 0.69 | 0.70 | 15.04 | 18.54 | 3.984 | **0.0002** | 0.0036 | 1 |
| RIFG[mIns] | 1.22 | 1.55 | 0.32 | 0.28 | 26.48 | 17.96 | -3.937 | **0.0003** | 0.0048 | 1 |
| RSM1 [NAA/mIns] | 5.83 | 4.57 | 1.29 | 1.21 | 22.13 | 26.57 | 3.589 | **0.0008** | 0.0060 | 1 |
| OCC [NAA/mIns] | 5.40 | 4.59 | 0.90 | 0.84 | 16.59 | 18.22 | 3.304 | **0.0018** | 0.0071 | 1 |
| LSM1[Cho] | 0.47 | 0.53 | 0.06 | 0.09 | 12.79 | 17.04 | -3.062 | **0.0036** | 0.0083 | 1 |
| OCC[NAA] | 6.34 | 5.83 | 0.59 | 0.61 | 9.31 | 10.54 | 3.056 | **0.0036** | 0.0095 | 1 |
| LSM1[mIns] | 1.09 | 1.30 | 0.24 | 0.27 | 22.01 | 20.43 | -3.040 | **0.0038** | 0.0107 | 1 |
| RSTR[Cho] | 0.59 | 0.67 | 0.09 | 0.12 | 15.26 | 17.29 | -2.948 | **0.0049** | 0.0119 | 1 |
| OCC[Glx] | 6.09 | 5.17 | 1.12 | 1.13 | 18.40 | 21.88 | 2.925 | **0.0052** | 0.0131 | 1 |
| LSTR [NAA/mIns] | 5.03 | 3.98 | 1.42 | 1.24 | 28.26 | 31.21 | 2.807 | **0.0071** | 0.0143 | 1 |
| PreSMA[mIns] | 1.32 | 1.56 | 0.25 | 0.36 | 19.08 | 22.92 | -2.716 | **0.0091** | 0.0155 | 1 |
| RIFG[Cho] | 0.57 | 0.65 | 0.10 | 0.10 | 16.61 | 15.45 | -2.627 | **0.0115** | 0.0167 | 1 |
| RSM1[Cho] | 0.48 | 0.53 | 0.05 | 0.08 | 10.63 | 14.88 | -2.624 | **0.0116** | 0.0179 | 1 |
| RSM1[NAA] | 6.46 | 6.08 | 0.40 | 0.65 | 6.17 | 10.65 | 2.494 | **0.0160** | 0.0190 | 1 |
| LSTR[Cho] | 0.62 | 0.69 | 0.09 | 0.11 | 15.34 | 16.11 | -2.433 | **0.0187** | 0.0202 | 1 |
| LSM1[NAA] | 6.22 | 5.83 | 0.40 | 0.72 | 6.50 | 12.28 | 2.411 | **0.0197** | 0.0214 | 1 |
| LSTR[NAA] | 6.25 | 5.78 | 0.85 | 0.61 | 13.60 | 10.51 | 2.286 | 0.0266 | 0.0226 | 0 |
| RIFG[Cr] | 5.71 | 6.12 | 0.64 | 0.70 | 11.15 | 11.43 | -2.171 | 0.0348 | 0.0238 | 0 |
| PreSMA[Cho] | 0.55 | 0.61 | 0.09 | 0.12 | 16.34 | 19.62 | -2.149 | 0.0366 | 0.0250 | 0 |
| RSM1[mIns] | 1.16 | 1.47 | 0.35 | 0.65 | 30.02 | 44.48 | -2.059 | 0.0448 | 0.0262 | 0 |
| preSMA[Cr] | 5.02 | 5.43 | 0.71 | 0.77 | 14.07 | 14.19 | -1.980 | 0.0533 | 0.0274 | 0 |
| RSTR[Glx] | 5.20 | 4.34 | 1.62 | 1.64 | 31.18 | 37.73 | 1.894 | 0.0641 | 0.0286 | 0 |
| LSM1[Cr] | 4.29 | 4.55 | 0.38 | 0.61 | 8.96 | 13.45 | -1.802 | 0.0777 | 0.0298 | 0 |
| LSTR[mIns] | 1.33 | 1.64 | 0.49 | 0.78 | 36.65 | 47.84 | -1.671 | 0.1012 | 0.0310 | 0 |
| OCC[mIns] | 1.19 | 1.30 | 0.18 | 0.30 | 14.92 | 22.94 | -1.662 | 0.1029 | 0.0321 | 0 |
| PreSMA[Glx] | 5.66 | 6.17 | 1.03 | 1.27 | 18.18 | 20.57 | -1.561 | 0.1249 | 0.0333 | 0 |
| RSM1[Cr] | 4.54 | 4.72 | 0.32 | 0.55 | 7.10 | 11.67 | -1.369 | 0.1772 | 0.0345 | 0 |
| RSTR[Cr] | 5.36 | 5.56 | 0.58 | 0.71 | 10.91 | 12.85 | -1.074 | 0.2882 | 0.0357 | 0 |
| LSTR[Glx] | 5.74 | 5.19 | 2.09 | 1.61 | 36.37 | 30.94 | 1.042 | 0.3023 | 0.0369 | 0 |
| RSTR[NAA] | 5.91 | 5.74 | 0.55 | 0.65 | 9.37 | 11.32 | 1.035 | 0.3056 | 0.0381 | 0 |
| RIFG[NAA] | 7.11 | 6.91 | 0.74 | 0.73 | 10.39 | 10.60 | 0.985 | 0.3293 | 0,0393 | 0 |
| PreSMA[NAA] | 5.95 | 5.77 | 0.65 | 0.84 | 10.84 | 14.55 | 0.875 | 0.3860 | 0.0405 | 0 |
| OCC[Cho] | 0.34 | 0.35 | 0.05 | 0.07 | 14.80 | 20.47 | -0.720 | 0.4748 | 0,0417 | 0 |
| RSM1[Glx] | 4.86 | 5.16 | 1.86 | 1.23 | 38.29 | 23.83 | -0.661 | 0.5115 | 0.0429 | 0 |
| LSRT[Cr] | 5.62 | 5.52 | 0.75 | 0.73 | 13.38 | 13.17 | 0.463 | 0.6458 | 0.0440 | 0 |
| RSTR [NAA/mIns] | 4.41 | 4.24 | 1.17 | 2.08 | 26.44 | 49.06 | 0.361 | 0.7193 | 0.0452 | 0 |
| RSTR[mIns] | 1.52 | 1.58 | 0.89 | 0.63 | 58.80 | 40.28 | -0.253 | 0.8010 | 0.0464 | 0 |
| RIFG[Glx] | 5.29 | 5.23 | 1.36 | 1.12 | 25.66 | 21.41 | 0.181 | 0.8570 | 0.0476 | 0 |
| OCC[Cr] | 4.86 | 4.88 | 0.35 | 0.42 | 7.26 | 8.71 | -0.128 | 0.8984 | 0.0488 | 0 |
| LSM1[Glx] | 4.29 | 4.26 | 0.82 | 0.95 | 19.14 | 22.17 | 0.112 | 0.9111 | 0.0500 | 0 |
|  |  |  |  |  |  |  |  |  |  |  |
| **(B)** | Mean YA | Mean OA | SD  YA | SD  OA | COV YA | COV OA | t(49) | p | Critical p-value | FDR test |
| RSM1 GM | 0.346 | 0.301 | 0.021 | 0.029 | 5.928 | 9.595 | 6.4821 | **0.0000** | 0.0036 | 1 |
| LSM1 GM | 0.317 | 0.266 | 0.048 | 0.029 | 15.248 | 10.878 | 4.6021 | **0.0000** | 0.0071 | 1 |
| RIFG GM | 0.542 | 0.498 | 0.043 | 0.043 | 7.974 | 8.582 | 3.6939 | **0.0006** | 0.0107 | 1 |
| OCC GM | 0.600 | 0.575 | 0.025 | 0.031 | 4.201 | 5.350 | 3.2674 | **0.0020** | 0.0143 | 1 |
| RSTR GM | 0.516 | 0.465 | 0.042 | 0.077 | 8.144 | 16.536 | 2.9139 | **0.0054** | 0.0179 | 1 |
| LSTR GM | 0.480 | 0.443 | 0.050 | 0.074 | 10.347 | 16.782 | 2.0807 | 0.0427 | 0.0214 | 0 |
| preSMA GM | 0.497 | 0.477 | 0.038 | 0.042 | 7.610 | 8.807 | 1.7757 | 0.0820 | 0.0250 | 0 |
| RSTR WM | 0.397 | 0.439 | 0.053 | 0.109 | 13.376 | 24.917 | -1.7172 | 0.0923 | 0.0286 | 0 |
| OCC WM | 0.318 | 0.335 | 0.038 | 0.033 | 12.086 | 9.989 | -1.6809 | 0.0992 | 0.0321 | 0 |
| RIFG WM | 0.341 | 0.376 | 0.074 | 0.075 | 21.613 | 19.872 | -1.6476 | 0.1058 | 0.0357 | 0 |
| RSM1 WM | 0.563 | 0.581 | 0.028 | 0.055 | 4.952 | 9.442 | -1.4643 | 0.1495 | 0.0393 | 0 |
| LSM1 WM | 0.618 | 0.636 | 0.057 | 0.057 | 9.201 | 8.986 | -1.1484 | 0.2564 | 0.0429 | 0 |
| preSMA WM | 0.321 | 0.304 | 0.055 | 0.056 | 17.275 | 18.311 | 1.0388 | 0.3040 | 0.0464 | 0 |
| LSTR WM | 0.453 | 0.473 | 0.066 | 0.097 | 14.601 | 20.454 | -0.8515 | 0.3986 | 0.0500 | 0 |

LSM1 = left sensorimotor cortex; LSTR = left striatum; OCC = occipital cortex; preSMA = pre-supplementary motor area; RIFG = right inferior frontal gyrus; RSTR = right striatum; RSM1 = right sensorimotor cortex. Data showed for young (YA, n=25) and older (OA, n=26) adults. A false discovery rate (FDR) controlling procedure was used to control for the probability of type I errors. The p-value should be lower than the critical p-value, as calculated with the FDR method, to survive multiple comparisons. The column FDR test represents significance after correction for multiple comparisons (1 = significant and 0 = not significant). Statistically significant group differences are highlighted in **bold**.

**Supplemental Table S2.** Results of the Pearson correlations between uncorrected neurometabolite variables and fractional tissue volumes of gray matter (GM) and white matter (WM) in the seven tested regions of interest (ROIs).

| ROI |  |  | n | NAA | Glx | Cr | Cho | mIns | NAA/mIns |
| --- | --- | --- | --- | --- | --- | --- | --- | --- | --- |
| LSM1 | GM | Young | 29 | **-0.564**** | -0.078 | **-0.403*** | -0.223 | -0.284 | 0.015 |
|  |  | Old | 29 | 0.027 | -0.170 | 0.157 | 0.167 | 0.048 | -0.100 |
|  | WM | Young | 29 | **0.613***** | 0.121 | **0.430*** | 0.202 | 0.267 | 0.023 |
|  |  | Old | 29 | **0.445*** | 0.277 | 0.297 | 0.209 | 0.249 | 0.044 |
| LSTR | GM | Young | 30 | 0.298 | 0.358 | 0.278 | 0.148 | **0.590***** | **-0.607***** |
|  |  | Old | 27 | -0.158 | -0.064 | -0.062 | -0.115 | 0.064 | -0.038 |
|  | WM | Young | 30 | -0.273 | **-0.415*** | -0.266 | -0.069 | **-0.555**** | **0.577**** |
|  |  | Old | 27 | 0.181 | 0.011 | 0.091 | 0.140 | -0.050 | 0.032 |
| OCC | GM | Young | 30 | **-0.374*** | -0.156 | -0.125 | -0.022 | -0.030 | -0.207 |
|  |  | Old | 28 | 0.339 | 0.352 | **0.481*** | 0.043 | 0.124 | 0.016 |
|  | WM | Young | 30 | **0.369*** | 0.149 | 0.118 | 0.117 | 0.047 | 0.174 |
|  |  | Old | 28 | -0.173 | -0.215 | -0.196 | 0.033 | **-0.380*** | 0.260 |
| preSMA | GM | Young | 30 | 0.238 | -0.052 | 0.219 | -0.083 | 0.290 | -0.192 |
|  |  | Old | 28 | 0.315 | 0.243 | 0.309 | 0.158 | **0.375*** | -0.274 |
|  | WM | Young | 30 | -0.015 | 0.165 | -0.135 | 0.067 | -0.161 | 0.195 |
|  |  | Old | 28 | 0.154 | 0.208 | 0.236 | 0.207 | -0.119 | **0.403*** |
| RIFG | GM | Young | 29 | -0.188 | 0.052 | -0.226 | -0.243 | 0.124 | -0.207 |
|  |  | Old | 28 | 0.099 | 0.119 | 0.029 | 0.052 | 0.174 | -0.083 |
|  | WM | Young | 29 | 0.095 | -0.191 | 0.163 | 0.048 | -0.186 | 0.217 |
|  |  | Old | 28 | 0.163 | 0.019 | 0.114 | 0.042 | -0.045 | 0.121 |
| RSTR | GM | Young | 30 | 0.285 | -0.145 | 0.342 | 0.307 | 0.277 | **-0.486**** |
|  |  | Old | 28 | 0.014 | 0.235 | 0.062 | -0.010 | -0.108 | 0.187 |
|  | WM | Young | 30 | -0.263 | 0.128 | -0.331 | -0.302 | -0.263 | **0.519**** |
|  |  | Old | 28 | 0.078 | -0.168 | 0.002 | 0.075 | 0.107 | -0.137 |
| RSM1 | GM | Young | 30 | -0.244 | -0.206 | **-0.437*** | -0.038 | -0.166 | 0.048 |
|  |  | Old | 27 | -0.102 | 0.024 | -0.008 | -0.145 | -0.079 | 0.145 |
|  | WM | Young | 30 | **0.444*** | 0.312 | **0.552**** | 0.142 | 0.309 | -0.104 |
|  |  | Old | 27 | 0.284 | 0.063 | 0.172 | 0.239 | 0.232 | -0.224 |

LSM1 = left sensorimotor cortex; LSTR = left striatum; OCC = occipital cortex; preSMA = pre-supplementary motor area; RIFG = right inferior frontal gyrus; RSTR = right striatum; RSM1 = right sensorimotor cortex; NAA = N-acetylaspartate; Glx = glutamate-glutamine complex; Cr = creatine + phosphocreatine; Cho = choline; mIns = myo-inositol; NAA/mIns = NAA to mIns ratio; *p < 0.05; ** p < 0.01; *** p < 0.001. Significant correlations at p < 0.05 are in **bold**.

**Supplemental Table S3.** Results of the Pearson correlations between tissue-corrected neurometabolite variables and GoRT. Data are presented separately for each of the two age groups.

| ROI |  | n | NAA | Glx | Cho | mIns | NAA/mIns |
| --- | --- | --- | --- | --- | --- | --- | --- |
| LSM1 | Young | 25 | 0.286 | 0.293 | 0.091 | 0.203 | -0.045 |
|  | Old | 26 | **0.510**** | -0.334 | 0.277 | 0.269 | 0.178 |
| LSTR | Young | 25 | **-0.468*** | **-0.572**** | **-0.418*** | **-0.410*** | 0.175 |
|  | Old | 26 | **0.480*** | 0.093 | -0.044 | -0.252 | 0.294 |
| OCC | Young | 25 | 0.272 | 0.134 | 0.181 | 0.051 | 0.187 |
|  | Old | 26 | 0.275 | -0.298 | -0.154 | 0.224 | -0.081 |
| preSMA | Young | 25 | 0.390‡ | -0.122 | -0.083 | 0.108 | 0.099 |
|  | Old | 26 | 0.128 | 0.024 | 0.082 | -0.037 | 0.125 |
| RIFG | Young | 25 | 0.143 | 0.056 | -0.096 | -0.087 | 0.003 |
|  | Old | 26 | -0.083 | -0.096 | -0.166 | -0.125 | 0.106 |
| RSTR | Young | 25 | 0.254 | **0.521**** | 0.137 | -0.286 | 0.139 |
|  | Old | 26 | -0.176 | 0.037 | -0.234 | 0.051 | -0.041 |
| RSM1 | Young | 25 | **0.421*** | 0.372 | 0.300 | 0.094 | 0.048 |
|  | Old | 26 | **0.412*** | -0.093 | 0.072 | **0.430*** | -0.361 |

LSM1 = left sensorimotor cortex; LSTR = left striatum; OCC = occipital cortex; preSMA = pre-supplementary motor area; RIFG = right inferior frontal gyrus; RSTR = right striatum; RSM1 = right sensorimotor cortex; NAA = N-acetylaspartate; Glx = glutamate-glutamine complex; Cho = choline; mIns = myo-inositol; NAA/mIns = NAA to mIns ratio; *p < 0.05; ** p < 0.01; *** p < 0.001; Significant correlations at p < 0.05 are in **bold**.

**Supplemental Table S4.** Results of the Pearson correlations between tissue-corrected neurometabolite variables and GoRT_40-20_. Data are presented separately for each of the two age groups.

| ROI |  | n | NAA | Glx | Cho | mIns | NAA/mIns |
| --- | --- | --- | --- | --- | --- | --- | --- |
| LSM1 | Young | 25 | 0.196 | -0.010 | 0.022 | 0.048 | 0.069 |
|  | Old | 26 | 0.173 | -0.017 | 0.371 | 0.334 | -0.287 |
| LSTR | Young | 25 | -0.075 | -0.079 | -0.191 | 0.028 | -0.110 |
|  | Old | 26 | -0.066 | -0.017 | -0.176 | 0.187 | -0.265 |
| OCC | Young | 25 | -0.149 | -0.071 | -0.047 | -0.208 | 0.134 |
|  | Old | 26 | 0.083 | 0.208 | 0.008 | 0.105 | -0.065 |
| preSMA | Young | 25 | -0.014 | -0.286 | -0.121 | 0.137 | -0.232 |
|  | Old | 26 | -0.063 | 0.043 | 0.142 | -0.057 | 0.013 |
| RIFG | Young | 25 | -0.243 | 0.113 | -0.289 | -0.017 | -0.169 |
|  | Old | 26 | 0.019 | 0.004 | -0.080 | -0.277 | 0.382‡ |
| RSTR | Young | 25 | 0.126 | **0.397*** | -0.233 | 0.045 | -0.114 |
|  | Old | 26 | -0.111 | -0.019 | -0.156 | -0.021 | 0.291 |
| RSM1 | Young | 25 | 0.200 | -0.009 | -0.162 | -0.066 | -0.056 |
|  | Old | 26 | 0.093 | -0.110 | 0.135 | 0.275 | -0.203 |

LSM1 = left sensorimotor cortex; LSTR = left striatum; OCC = occipital cortex; preSMA = pre-supplementary motor area; RIFG = right inferior frontal gyrus; RSTR = right striatum; RSM1 = right sensorimotor cortex; NAA = N-acetylaspartate; Glx = glutamate-glutamine complex; Cho = choline; mIns = myo-inositol; NAA/mIns = NAA to mIns ratio; *p < 0.05; ** p < 0.01; *** p < 0.001; ‡ p = 0.054. Significant correlations at p < 0.05 are in **bold**.

**Supplemental Table S5.** Results of the Pearson correlations between tissue-corrected neurometabolite variables and SSRT. Data are presented separately for each of the two age groups.

| ROI |  | n | NAA | Glx | Cho | mIns | NAA/mIns |
| --- | --- | --- | --- | --- | --- | --- | --- |
| LSM1 | Young | 25 | -0.280 | -0.338 | -0.139 | -0.020 | -0.091 |
|  | Old | 26 | 0.250 | 0.035 | 0.155 | 0.288 | -0.161 |
| LSTR | Young | 25 | 0.031 | -0.047 | 0.132 | 0.029 | -0.091 |
|  | Old | 26 | 0.315 | -0.047 | 0.133 | -0.081 | 0.109 |
| OCC | Young | 25 | 0.134 | -0.033 | 0.043 | 0.345 | -0.265 |
|  | Old | 26 | 0.292 | 0.151 | -0.335 | **0.584**** | **-0.465*** |
| preSMA | Young | 25 | -0.273 | -0.019 | -0.301 | 0.226 | **-0.474*** |
|  | Old | 26 | -0.040 | 0.116 | 0.074 | -0.078 | 0.055 |
| RIFG | Young | 25 | -0.214 | -0.138 | -0.187 | -0.174 | 0.123 |
|  | Old | 26 | 0.127 | 0.129 | 0.185 | 0.234 | -0.190 |
| RSTR | Young | 25 | 0.029 | -0.229 | 0.060 | -0.119 | 0.165 |
|  | Old | 26 | 0.076 | -0.070 | 0.109 | -0.108 | 0.000 |
| RSM1 | Young | 25 | 0.372 | 0.296 | -0.059 | 0.332 | -0.128 |
|  | Old | 26 | **0.457*** | 0.280 | **0.470*** | -0.013 | 0.166 |

LSM1 = left sensorimotor cortex; LSTR = left striatum; OCC = occipital cortex; preSMA = pre-supplementary motor area; RIFG = right inferior frontal gyrus; RSTR = right striatum; RSM1 = right sensorimotor cortex; NAA = N-acetylaspartate; Glx = glutamate-glutamine complex; Cho = choline; mIns = myo-inositol; NAA/mIns = NAA to mIns ratio; *p < 0.05; ** p < 0.01; *** p < 0.001; Significant correlations at p < 0.05 are in **bold**.

**Supplemental Table S6.** Multiple linear regression model summary for ^1^H-MRS neurometabolite predictors of GoRT (young and old) and SSRT (old). Directions of associations between performance measures and models’ principal neurometabolite predictors (shaded in gray; similar to Table 3 in the manuscript) are indicted: (↑) for positive association and (↓) for negative association. ‡R^2^ change represent the he amount by which R­^2^ is reduced if a particular independent variable is removed from the model.

| Performance  /Age group | R^2^ | R^2^-Adj. | F | Contributing neurometabolite | R^2^ change‡ | p-value |
| --- | --- | --- | --- | --- | --- | --- |
| **GoRT YA** | 0.619 | 0.492 | 4.874** |  |  |  |
|  |  |  |  | RSM1 [NAA] ↑ | 0.133 | 0.022 |
|  |  |  |  | RSTR [Glx] ↑ | 0.109 | 0.036 |
|  |  |  |  | LSTR [Glx] ↓ | 0.080 | 0.068 |
|  |  |  |  | LSTR [mIns] | 0.017 | 0.388 |
|  |  |  |  | LSTR [Cho] | 0.013 | 0.442 |
|  |  |  |  | LSRT [NAA] | 0.002 | 0.498 |
| **GoRT OA** | 0.399 | 0.284 | 3.485* |  |  |  |
|  |  |  |  | LSTR [NAA] ↑ | 0.063 | 0.153 |
|  |  |  |  | LSM1 [NAA] ↑ | 0.053 | 0.188 |
|  |  |  |  | RSM1 [mIns] ↑ | 0.051 | 0.195 |
|  |  |  |  | RSM1 [NAA] | 0.000 | 0.986 |
| **SSRT OA** | 0.464 | 0.362 | 4.553** |  |  |  |
|  |  |  |  | RSM1 [NAA] ↑ | 0.072 | 0.107 |
|  |  |  |  | OCC [mIns] ↑ | 0.051 | 0.171 |
|  |  |  |  | OCC [NAA/mIns] | 0.002 | 0.790 |
|  |  |  |  | RSM1 [Cho] | 0.000 | 0.971 |

Multiple regression model R^2^. Adjusted R^2^ (R^2^-Adj). and F-value (p-levels: *p < 0.05; **p < 0.01).

Abbreviations: NAA = N-acetylaspartate; Glx = glutamate-glutamine complex; Cho = choline; mIns = myo-inositol; RSM1 = right sensorimotor cortex; RSTR = right striatum; LSM1 = left sensorimotor cortex; LSTR = left striatum; OCC = occipital cortex. Neurometabolite levels were corrected for voxel tissue composition.
